# Association of sirtuin 1 gene polymorphisms with nephrolithiasis in Eastern chinese population

**DOI:** 10.1080/0886022X.2019.1568258

**Published:** 2019-02-02

**Authors:** Jiebin Hou, Jiarong Ding, Lu Li, Yonghan Peng, Xiaofeng Gao, Zhiyong Guo

**Affiliations:** a Department of Nephrology, Shanghai Changhai Hospital, Shanghai, China;; b Department of Urology, Shanghai Changhai Hospital, Shanghai, China

**Keywords:** SIRT1, single nucleotide polymorphism, kidney stone, CaOx crystal, Chinese

## Abstract

Sirtuin 1 (SIRT1), an NAD^+^-dependent deacylase, has been identified to be associated with renal tubular inflammatory conditions and metabolic disorders, which are risk factors of nephrolithiasis. To further confirm the role of the SIRT1 in kidney stone formation, the expression of SIRT1 was analyzed based on a mouse model and the genetic polymorphisms of *SIRT1* gene was compared between patients with kidney stones and controls. The calcium oxalate (CaOx) crystal-induced renal injury model was established to analyzed the expression of SIRT1 in the kidney tissue of both wild-type and ApoE(−/−) mice. And a total of 430 Eastern Chinese subjects (215 patients with nephrolithiasis and 215 age- and gender-matched controls) were recruited for the present study to investigate the associations between 6 common single nucleotide polymorphisms (SNPs) (i.e., rs10509291, rs3740051, rs932658, rs33957861, rs3818292 and rs1467568) in the *SIRT1* gene and the incidence of kidney stones. Pairwise linkage disequilibrium and the haplotypes of the 6 SNPs were also analyzed. The genotypes of SIRT1 gene polymorphisms were analyzed by a Snapshot assay. Reduced expression of SIRT1 was observed in the kidney of the mice in the crystal group, revealing the potential role of SIRT1 in the nephrolithiasis. However, we did not find a significant association between the 6 SNPs of the SIRT1 gene and kidney stone formation in the Eastern Chinese population.

## 1. Introduction

The worldwide prevalence of kidney stone disease, also known as nephrolithiasis, has increased in recent years from 5% to 12% [1,2], endangering the health of the public and the quality of life of patients. The formation of kidney stones may lead to a decline in renal function [3] and even persistent kidney damage, thereby increasing the risk of acute or chronic kidney disease, especially after recurrent attacks [4]. Furthermore, kidney stones are indicated to be associated with cardiovascular diseases including coronary heart disease and stroke [5,6]. Therefore, nephrolithiasis is increasingly recognized as a systemic disorder.

Approximately 70% of stones are composed of calcium oxalate (CaOx); other contents include calcium phosphate, uric acid and other salts [7]. Most calcium oxalate (CaOx) stones develop from sub-epithelial plaques of calcium phosphate (CaP), known as Randall's plaques, which are also scattered in the interstitium and around the collecting ducts and blood vessels [8]. In addition to abnormal mineral metabolism, oxidative stress (OS), inflammation and aberrant crystallization inhibition play significant roles in the deposition of CaP in the basement membrane of renal tubules or vessels [9]. This plaque deposition further aggravates OS, causing injury to tubular epithelial cells. Renal injury and the normal aging process are proposed to promote this process. With the aggregation and calcification of plaques, oxidative and inflammatory reactions subsequently promote the further formation of stones [10]. Cell injury, inflammation, OS, interstitial fibrosis and intratubular crystal deposition have been observed in renal biopsies during stone formation at all stages and promote the overall process [11,12]. According to research, the pathophysiology of urinary stone formation is complex and involves metabolic, genetic, and environmental factors [13].

As a protein member of a conserved family of NAD(+)-dependent deacylases called sirtuins, sirtuin1 (SIRT1) is associated with numerous cellular signaling pathways that are mainly involved in cytoprotective effects and metabolic regulation [14]. Increasing evidence shows that reduced SIRT1 levels are closely associated with various inflammatory diseases. Therefore, pharmacologic activation of SIRT1 could be a promising therapeutic strategy for OS- or inflammation-related diseases [15]. In the kidneys, SIRT1 may inhibit OS, inflammation and renal cell apoptosis. The renal protective effects of SIRT1 have been found in various models of renal disorders, including diabetic nephropathy, acute kidney injury, chronic kidney disease and lupus nephritis. In cisplatin-induced renal injury, SIRT1 inhibits oxidative stress by upregulating catalase expression and increasing the number and functions of mitochondria [16,17]. In addition to protective effects in tubular cells, SIRT1 also exhibits antioxidative and anti-apoptosis activity in vascular endothelial cells [18]. Considering that vessels and tubules under conditions of OS represent the origins of plaques, we proposed a potential association between SIRT1 and the pathogenesis of nephrolithiasis. In addition, kidney fibrogenesis was observed with the calcification and formation of calcium crystals in the kidney (Hu et al., 2015), and SIRT1 up-regulation induced by an SIRT1 activator can attenuate renal fibrosis and renal injury [19,20]. Therefore, SIRT1 is considered a new potential therapeutic target for kidney stones and warrants further investigation.

Understanding the genetic basis of complicated human diseases such as nephrolithiasis has been increasingly emphasized in medical research. In this study, the role of *SIRT1* gene in nephrolithiasis was firstly explored based on a mouse model of CaOx crystal-induced renal injury. In addition, genetic variations of the *SIRT1* gene have been found to be associated with cardiovascular diseases [21] and chronic inflammatory states [22]. To confirm whether there is a correlation between *SIRT1* and the risk of kidney stones, the associations between single nucleotide polymorphisms (SNPs) of the *SIRT1* gene and the incidence of nephrolithiasis in the patients were investigated in the present study.

## 2. Material and methods

### 2.1. Experimental animals

Twelve wild-type male C57BL/6 mice (7–8 weeks old) were purchased from the Shanghai SLAC Animal Co., Ltd. (Shanghai, China). Twelve male ApoE KO (ApoE–/–) mice of the same age were acquired from the Shanghai Institute of Materia Medica, Chinese Academy of Sciences. After conditioned housing for one week, wild-type and ApoE KO mice were respectively and equally divided into the control and the crystal model groups with 6 mice each. The crystal group was administered glyoxylate (100 mg/kg/day) by intraperitoneal injections for 3 consecutive days, and the control group was intra-abdominally injected daily with normal-volume saline (20 mL/kg/day) per day as previously described [23]. On day 3 after the administration of glyoxalate, kidney samples were collected, and the cortex and medulla junction tissue were dissected to be further analyzed. All animal studies were performed in accordance with the National Institutes of Health (NIH) guide for the Care and Use of Laboratory Animals. The experimental procedures were approved by the Ethical Committee for the Experimental Use of Animals at Second Military Medical University (Shanghai, China).

### 2.2. Western blot

Western blotting was conducted following previous method [23]. Harvested corticomedullary tissues from different groups were respectively homogenized in lysis buffer containing protease inhibitor and phosphatase inhibitor. The homogenates were centrifuged at 12,000 rpm for 5 min at 4 °C and the supernatant was collected. The protein concentration in each lysate was determined using a BCA protein assay kit (Thermo Fisher Scientific). Equal amount of each sample was subjected to SDS-PAGE gel for separation and transferred onto a nitrocellulose membrane (GE Healthcare Life Sciences). After blocking, the membrane was incubated with rabbit polyclonal anti-SIRT1 antibodies (1:1000, Abcam) at 4 °C overnight. After washing with TBST, the membrane was incubated with a fluorescence-conjugated secondary anti-rabbit antibody (1:10000, Licor) for 60 min at room temperature. The signals were visualized using the Odyssey Infrared Imaging System (Licor, NE, USA) and quantitatively analyzed by normalizing to β-actin using the Image J software (National Institutes of Health, Bethesda, MD, USA).

### 2.3. Clinical subjects

Here, we report a hospital-based case-control study with 215 patients who underwent urolithiasis surgery for kidney stones from eastern China at Changhai Hospital in Shanghai. The diagnosis of stones was confirmed by plain X-ray film and renal ultrasound. After surgery, the composition of the extracted stones was analyzed by chemical tests. The mineral compositions of these stones were determined by Fourier transform infrared spectroscopy. In our study, only patients with calcium-containing kidney stones, which are mainly composed of CaOx (>60%) were included. Demographic and clinical information was collected from all subjects, including age, gender, body mass index (BMI) and comorbidity information such as diabetes mellitus, hypertension and hyperlipidemia. The matched controls consisted of 215 healthy subjects admitted for routine health examinations in the same hospital. Ultrasonographic examinations were also performed for the controls to confirm the absence of renal stones. All subjects were of Eastern Chinese descent. The study protocol was approved by the Ethical Committee of Changhai Hospital (Shanghai, China). And informed consent was obtained from all patients and control subjects after a full explanation of the study.

### 2.4. SNP selection

Haploview 4.2 (Broad Institute of MIT and Harvard, Cambridge, MA, USA) was used to select the 6 tagging SNPs (i.e., rs10509291, rs3740051, rs932658, rs33957861, rs3818292 and rs1467568) in the *SIRT1* gene according to the Han Chinese database loaded from 1000 Genome Browers (https://www.ncbi.nlm.nih.gov/variation/tools/000genomes/) using the threshold of r^2^ >0.8 and a minor allele frequency (MAF) of >0.10.

### 2.5. Genotyping methods

Genomic DNA was isolated from peripheral blood leukocytes using the phenol–chloroform method. Genotyping of the six SNPs was performed using Snapshot technology. All experimental manipulations were completed in the Center for Human Genetics Research, Genesky Biotechnologies Inc. (Shanghai, China). Primers were designed with online Primer 3 software (http://frodo.wi.mit.edu). The corresponding primers used for each SNP in our study are listed in Table 1. Polymerase chain reaction (PCR) products were obtained by QIAGEN Company HotStar Taq multiple PCR and purified by shrimp alkali enzyme (SAP) (Promega, Madison, WI, USA) and external enzyme (exobiology I; Epicentre, Madison, WI, USA). Purified products were extended with the ABI Snapshot Multiplex kit. The reaction was performed under the following conditions: after denaturation at 96 °C for 1 min, 28 cycles of DNA amplification were performed using Taq PCR for 10 s at 96 °C (denaturation), 5 s at 55 °C (annealing), and 30 s at 60 °C (extension). Extension products were sampled with SAP on the ABI3130xl genetic sequence analyzer after purification. The final data were analyzed using GeneMapper 4.1 (Applied Biosystems, Foster City, CA, USA).

**Table 1.** Primers sequences.

SNP	Alleles	Location	PCR forward primers	PCR reverse primers
rs10509291	A/T	5′-flanking	GCCACTTGTGTCCTATTTTCTGTGAA	CAGAGGAGTGATTTTCACATTGGTTTT
rs3740051	G/A	5′-flanking	GAGGGGAAAAAAGCAACCGACTA	CTGGCCTGCCTTAGCCTTGTCT
rs932658	A/C	5′-flanking	GGCGAATTTGGCTGCACTACAC	GCGGGAGATTTAAACCCCATCA
rs33957861	C/T	intron1	CGCTCCCGGCTATCTTTTCTGT	GGGGTCAGCTTGCACTGTTCAT
rs3818292	G/A	intron5	GCTGACTGCCATCGAGAAGTGG	TGCATGCAACTGCAGCATCTTT
rs1467568	G/A	intron8	CCAGTGTGGTAACCAGGCTTTTG	CAGGCCCCAMGACCCACCTA
Primers of extension				
rs10509291	TTTTTTTTTTTTTTTTTTTTTTTTTTTTTTTTTTTTTTTTTTTTTCAGCAGAATCCACCCACTGA			
rs3740051	TTTTTTTCTCCTTTTGCCTCTCTTCCTACTT			
rs932658	TTTTTTTTTTTTTTTTTTTTTTTTTTTTTTTTTTTTTTTTTTTACACGCTCGCCAMAAAGAGG			
rs33957861	GGAATTCTGCTCACTCAGTTTCA			
rs3818292	TTTTTTTTTTTTTTTTTTTCCTTGACAGTTAATTATAGAAAACCTCAGAT			
rs1467568	TTTTTTTTTTTTTTTTTTCCTACTCTTTCACTTAAAMCCCAA			

### 2.6. Statistical analysis

Descriptive parameters are presented as the mean ± SD and categorical variables are presented using frequency. Statistical analyses were performed using SPSS 19.0 (SPSS, Chicago, IL, USA). The differences between two groups of mouse model were analyzed using Student’s t-test. The Hardy-Weinberg equilibrium test was performed in the controls for each variant before the association analysis of patients. SNPs that failed this test (*p* < 0.05 in the controls) were excluded. Pairwise linkage disequilibrium including |D’| and r^2^ was estimated and the corresponding maps were created using Haploview (version 4.2) (15). Pearson’s Chi-square test (χ2) was used to compare categorical variables between the groups. A logistic regression analysis was performed to calculate genotype- and allele-specific odds ratios (ORs) with 95% confidence intervals (CIs) after adjusting for age and sex as covariates. Haplotype frequencies among the SNPs were analyzed using PLINK version 1.07 (http://zzz.bwh.harvard.edu/plink/). In all tests, two-tailed values of *p* < 0.05 were considered statistically significant.

## 3. Results

### 3.1. Reduced expression of SIRT1 in the mice of crystal group

**Figure 1.**
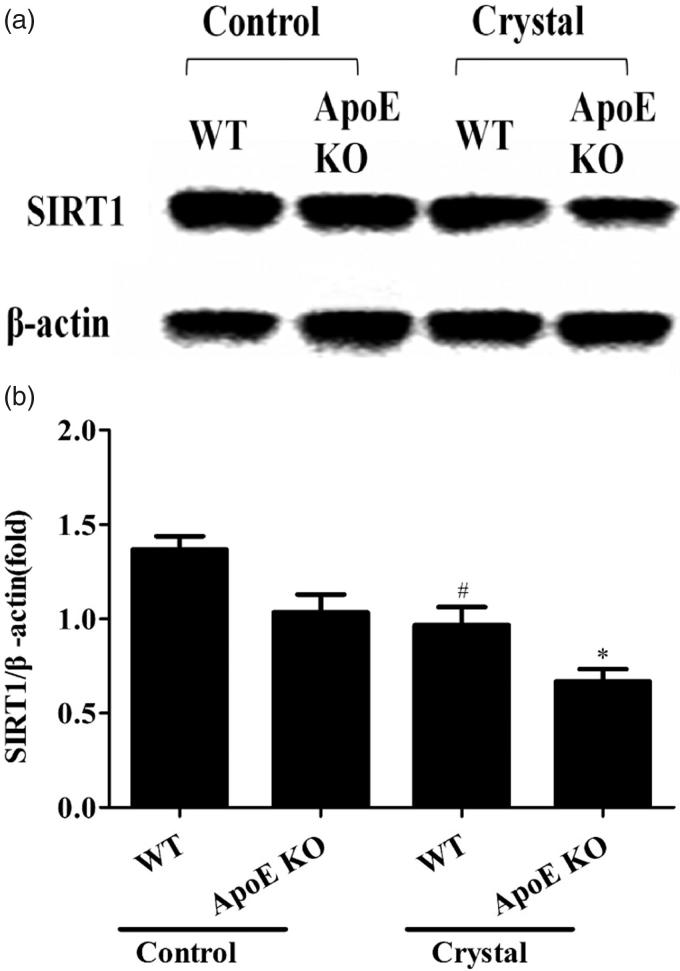
The expression of SIRT1 in the kidney tissue of mice. Western blot for SIRT1 protein in the corticomedullary tissue of kidney were analyzed based on both wild -type(WT) mice and ApoE KO mice. β-actin was used as loading control. Data were expressed as mean ± SEM (n = 6). *compared with the WT mice in the control group, *P* < 0.05; ^#^compared with the ApoE KO mice in the control group, *P* < 0.05.

As could be seen from Figure 1, the expression of SIRT1 were significantly decreased in the kidney tissue of crystal groups compared to the controls based on both wild-type and ApoE KO mice. And the difference between the expression of SIRT1 in wild-type and ApoE KO was not significant.

### 3.2. Demographic characteristics of the patients and controls

In total, 430 subjects participated in this study, including 215 patients with calcium-containing kidney stones and 215 age- and sex-matched controls. The baseline characteristics and clinical data of the patients and controls are presented in Table 2. Males accounted for 123 (57.2%) subjects in both groups. The average age of the patients with nephrolithiasis was 46.85 years, which was not significantly different from that of the controls. BMI was not significantly different between the groups, with a value of 23.87 ± 3.13 kg/m^2^ for the nephrolithiasis group compared to 23.39 ± 1.96 kg/m^2^ for the control group. No significant differences were noted in the distribution of hypertension, diabetes mellitus (DM) or hyperlipoidemia in the two groups.

**Table 2.** Demographic and characteristics of the nephrolithiasis patients and controls.

Characteristics	Controls(n = 215)	Nephrolithiasis patients (n = 215)	*P*
Gender (Male/Female)	123/92	123/92	1.000
Age (years)	46.85 ± 12.56	45.86 ± 8.76	0.343
BMI (Kg/m^2)^	23.39 ± 1.96	23.87 ± 3.13	0.057
Obesity No.	7	8	1.000
Hypertension No.	96	109	0.247
DM No.	61	65	0.751
Hyperlipemia No.	27	29	0.886

### 3.3. Distributions of alleles and genotype frequencies

Six SNPs (i.e., rs10509291, rs3740051, rs932658, rs33957861, rs3818292 and rs1467568) of the *SIRT1* gene were selected as tags for analysis. The genotypic distribution of each SNP was consistent with HWE for the control group (*p* = 0.368, 0.368, 0.052, 0.227, 0.368 and 0.052, respectively). The frequency distributions of each SNP genotype and allele in the patients and controls are summarized in Table 3. No significant association was observed between nephrolithiasis and genotypic distribution in the two groups by the Chi-square test. Furthermore, the logistic regression analysis with adjustments for age and gender also revealed no significant risk association between nephrolithiasis and each SNP.

**Table 3.** Genotypic distribution of the SIRT1 gene variants.

SNP sites	case	controls	P*	OR†	*P*†
**rs10509291**					
Genotype					
TT	114 (53.0%)	123 (57.2%)	0.215	1	
TA	91 (42.3%)	76 (35.3%)		1.32 (0.88-1.97)	0.175
AA	10 (4.7%)	16 (7.4%)		0.67 (0.29-1.55)	0.343
Allele					
T	319 (74.2%)	322 (74.9%)	0.812	1	
A	111 (25.8%)	108 (25.1%)		1.05 (0.77-1.43)	0.780
**rs3740051**					
Genotype					
AA	117 (54.4%)	123 (57.2%)	0.299	1	
GA	88 (40.9%)	76 (35.3%)		1.24 (0.83-1.85)	0.288
GG	10 (4.7%)	16 (7.4%)		0.65 (0.28-1.50)	0.321
Allele					
A	322 (74.9%)	322 (74.9%)	1.000	1	
G	108 (25.1%)	108 (25.1%)		1.01 (0.74-1.38)	0.965
**rs932658**					
Genotype					
AA	154 (71.6%)	145 (67.4%)	0.414	1	
CA	57 (26.5%)	68 (31.6%)		0.79 (0.52-1.20)	0.259
CC	4 (1.9%)	2 (0.9%)		1.88 (0.34-10.449)	0.473
Allele					
A	365 (84.9%)	358 (83.3%)	0.496	1	
C	65 (15.1%)	72 (16.7%)		0.87 (0.59-1.28)	0.483
**rs33957861**					
Genotype					
CC	171 (79.6%)	175 (81.4%)	0.200	1	
CT	40 (18.6%)	40 (18.6%)		1.03 (0.63-1.67)	0.920
TT	4 (1.9%)	0 (0.0%)		/	0.999
Allele					
C	382 (88.8%)	390 (90.7%)	0.366	1	
T	48 (11.2%)	40 (9.3%)		1.23 (0.79-1.92)	0.362
**rs3818292**					
Genotype					
AA	114 (53.0%)	123 (57.2%)	0.211	1	
GA	91 (42.3%)	76 (35.3%)		1.32 (0.88-1.97)	0.175
GG	10 (4.7%)	16 (7.4%)		0.67 (0.29-1.54)	0.343
Allele					
A	319 (74.2%)	322 (74.9%)	0.812	1	
G	111 (25.8%)	108 (25.1%)		1.05 (0.77-1.43)	0.780
**rs1467568**					
Genotype					
AA	154 (71.6%)	145 (67.4%)	0.585	1	
GA	58 (27.0%)	68 (31.6%)		0.80 (0.53-1.22)	0.295
GG	3 (1.4%)	2 (0.9%)		1.40 (0.23-8.54)	0.720
Allele					
A	366 (85.1%)	358 (83.3%)	0.431	1	
G	64 (14.9%)	72 (16.7%)		0.85 (0.58-1.26)	0.419

* Chi-square test.

† Logistic regression analysis after adjustment with age and sex.

**Figure 2.**
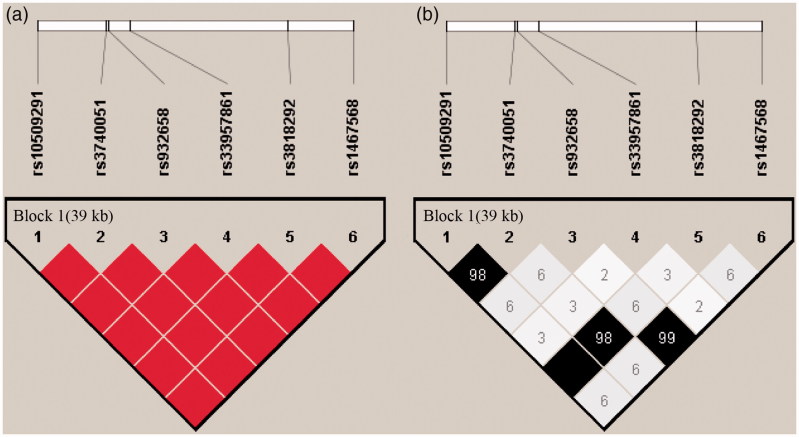
Linkage disequilibrium maps for SNPs genotyped in SIRT1 region. (A) Shades of red demonstrate the strength of the pairwise linkage disequilibrium based on D’, and numbers represent the value of D’ expressed as a percentage. The blanks represent D′=1. (B) Shades of gray show the strength of the pairwise linkage disequilibrium based on r^2^, and numbers indicate the value of r^2^ expressed as a percentage.

Pairwise linkage disequilibrium indicated that these 6 SNPs exhibited obvious linkage disequilibrium in one haplotype block (rs10509291, rs3740051, rs932658, rs33957861, rs3818292 and rs1467568) in this region (Figure 2). In the haplotype analysis (Table 4), by comparing the distribution frequencies between the patients and control subjects, we found no significant differences in effects for any haplotypes in *SIRT1*.

**Table 4.** Associations of one haplotype in SIRT1 region with kidney stone.

	Haplotype frequencies		
Haplotype	Nephrolithiasis	Control	P value
Block*			
TACCAG	0.150	0.167	0.491
AGACGA	0.254	0.251	0.937
TAATAA	0.112	0.099	0.370
TAACAA	0.485	0.488	0.914

* rs10509291; rs3740051; rs932658; rs33957861; rs3818292; rs1467568.

## 4. Discussion

The human *SIRT1* gene is located in the chromosome region 10q21.3 [24] and its alternative splicing results in multiple transcript variants. As a human sirtuin, SIRT1 may function as an intracellular regulatory protein via NAD^+^-dependent deacetylase activity. SIRT1 has been observed in both the nucleus and the cytoplasm where it interacts with nuclear and cytosolic proteins in different roles [25]. SIRT1 is expressed in a wide range of tissues and organs in humans, with relatively high expression levels in the fat, kidneys and liver [26]. SIRT1 protects cells by regulating metabolism and exerting anti-apoptotic, anti-oxidative and anti-inflammatory effects against injury in renal cells and in cells from other tissues. The role of SIRT1 in the renal interstitial fibrosis have been concentrated these years. Tubulointerstitial fibrosis and decreased Sirt1 expression was found in the kidney of unilateral ureteral obstruction rats [27]. And the activation of SIRT1 was demonstrated to attenuate the inflammation and inhibit the oxidative stress in renal injury [28,29]. In present study, the association between nephrolithiasis and the expression of SIRT1 was firstly explored based on the mouse model of CaOx crystal-induced renal injury. According to previous researches, the level of SIRT1 could be regulated by the type or the knockout of ApoE [30,31]. But the different expression of SIRT1 in kidney was not found between the wild-type mice and the ApoE KO mice in this study. The reduced expression of SIRT1 in the crystal group was both observed in the wild-type mice and the ApoE KO mice, revealing the potential role of SIRT1 in the nephrolithiasis.

Given the accumulating evidence indicating the close association between SIRT1 and various diseases, genetic variations of *SIRT1* have been widely investigated. Several genetic polymorphisms of *SIRT1* have also been shown to be associated with different health conditions, such as metabolic disorders (obesity, hyperglycemia and hypertension) [21,32,33] and chronic inflammatory states [22]. Systemic inflammation [34], urine supersaturation [35] and renal injury induced by metabolic disorders may promote the formation of stones. However, the correlation between *SIRT1* polymorphisms and nephrolithiasis has not been studied.

Several genetic polymorphisms have been found to play important roles in the pathogenesis of calcium nephrolithiasis, including SNPs in transient receptor potential vanilloid member 5 (TRPV5) [36], calcitonin receptor gene (CALCR) [37] and vitamin D receptor (VDR) [38], which are related to the maintenance of calcium homeostasis or the matrix protein. SIRT1 enzymatically potentiates 1,25-dihydroxyvitamin D3 signaling via VDR deacetylation and is also considered a positive regulator of osteoblasts [39] and osteogenic differentiation [40]. Given the important role of *SIRT1* gene in calcium metabolism, genetic polymorphisms of *SIRT1* are considered potential impact factors for hypercalciuria, which leads to nephrolithiasis [41].

In this study, 6 common non-coding SNPs of *SIRT1* were selected as tags for analysis to explore the relationship between the *SIRT1* gene and kidney stone formation. Among them, the rs10509291, rs3740051, and rs932658 polymorphisms of the *SIRT1* gene were 5'-flanking and primarily function in the regulation of gene transcription. The rs33957861 (intron1), rs3818292 (intron5) and rs1467568 (intron8) polymorphisms are *SIRT1* gene intron variants. These 6 polymorphisms of SIRT1 have been widely investigated mainly in the context of metabolism and have been found to be associated with the risk of obesity (rs10509291, rs33957861, and rs1467568) [32,42,43] and carbohydrate metabolism disturbances (rs10509291 and rs1467568) [44,45]. In this study, the distribution of obesity, hypertension, diabetes and hyperlipidemia was matched between control and patient samples, with no significant differences. Therefore, we could focus on the effects of *SIRT1* gene in nephrolithiasis locally.

According to previous research, rs3740051 has been widely studied and its polymorphism was found to be associated with the expression of SIRT1. Female cancer patients carrying the G minor allele of rs3740051 exhibited higher serum SIRT1 levels [46]. Given the beneficial role of SIRT1 in oxidative reactions and cell injuries [47], the G allele for rs3740051, which elicits high SIRT1 levels, was hypothesized as a protective factor. However, the frequency of the genotype with the G allele in the stone group was nearly the same as that of the control group in our study. More studies are needed to explore whether SNPs are correlated with the expression of SIRT1 in kidney tissues. Rs3740051 and rs1467568 have been indicated to be associated with carotid atherosclerosis, especially in women [48]. The variant of rs932658 reportedly alters the transcriptional activities of the *SIRT1* gene promoter and changes SIRT1 levels, contributing to hernia development. This phenomenon was attributed to the regulatory roles of *SIRT1* gene in the differentiation of human cells [49]. In general, SNPs of *SIRT1* play different and complicated roles in different diseases. A direct effect of *SIRT1* gene in kidney stone formation was not found in the present study.

In conclusion, the reduced expression of SIRT1 was observed in the injured kidney of the mice induced by CaOx crystal, revealing the potential role of SIRT1 in the nephrolithiasis. In addition, we genetically analyzed 6 SNPs of the *SIRT1* gene in kidney stone patients and controls. However, no significant association was found between genetic polymorphisms and the development of nephrolithiasis. Participants in our study were of Eastern Chinese descent. Due to racial differences, various altered genotype distributions may be present in other populations. Further studies with larger sample sizes are required to explore the potential relationship between SIRT1 and nephrolithiasis.

## References

[1] Scales CD Jr., Smith AC, Hanley JM, et al. Prevalence of kidney stones in the United States. Eur Urol. 2012;62:160–165. 22498635 10.1016/j.eururo.2012.03.052 PMC3362665

[2] Ordon M, Urbach D, Mamdani M, et al. A population based study of the changing demographics of patients undergoing definitive treatment for kidney stone disease. J Urol. 2015;193:869–874. 25261806 10.1016/j.juro.2014.09.096

[3] Haley WE, Enders FT, Vaughan LE, et al. Kidney Function After the First Kidney Stone Event. Mayo Clin Proc. 2016;91:1744–1752. 27776839 10.1016/j.mayocp.2016.08.014 PMC5140038

[4] Sigurjonsdottir VK, Runolfsdottir HL, Indridason OS, et al. Impact of nephrolithiasis on kidney function. BMC Nephrol. 2015;16:149 26316205 10.1186/s12882-015-0126-1 PMC4551564

[5] Ando R, Nagaya T, Suzuki S, et al. Kidney stone formation is positively associated with conventional risk factors for coronary heart disease in Japanese men. J Urol. 2013;189:1340–1346. 23159273 10.1016/j.juro.2012.11.045

[6] Alexander RT, Hemmelgarn BR, Wiebe N, et al. Kidney stones and cardiovascular events: a cohort study. Clin J Am Soc Nephrol. 2014;9:506–512. 24311706 10.2215/CJN.04960513 PMC3944758

[7] Spivacow FR, Del Valle EE, Lores E, et al. Kidney stones: Composition, frequency and relation to metabolic diagnosis. Medicina (B Aires). 2016;76:343–348. 27959841

[8] Sepe V, Adamo G, La Fianza A, et al. Henle loop basement membrane as initial site for Randall plaque formation. Am J Kidney Dis. 2006;48:706–711. 17059989 10.1053/j.ajkd.2006.07.021

[9] Taylor ER, Stoller ML Vascular theory of the formation of Randall plaques. Urolithiasis 2015;43 (Suppl 1): 41–45. 10.1007/s00240-014-0718-4 25475492

[10] Khan SR Reactive oxygen species, inflammation and calcium oxalate nephrolithiasis. Transl Androl Urol 2014;3:256–276. 25383321 10.3978/j.issn.2223-4683.2014.06.04 PMC4220551

[11] Khan SR, Canales BK Unified theory on the pathogenesis of Randall's plaques and plugs. Urolithiasis. 2015;43 (Suppl 1):109–123. 25119506 10.1007/s00240-014-0705-9 PMC4373525

[12] Gan QZ, Sun XY, Ouyang JM Adhesion and internalization differences of COM nanocrystals on Vero cells before and after cell damage. Mater Sci Eng C Mater Biol Appl. 2016;59:286–295. 26652375 10.1016/j.msec.2015.10.012

[13] Shadman A, Bastani B Kidney Calculi: Pathophysiology and as a Systemic Disorder. Iran J Kidney Dis. 2017;11:180–191. 28575878

[14] Chang HC, Guarente L SIRT1 and other sirtuins in metabolism. Trends Endocrinol Metab. 2014;25:138–145. 24388149 10.1016/j.tem.2013.12.001 PMC3943707

[15] Xie J, Zhang X, Zhang L Negative regulation of inflammation by SIRT1. Pharmacol Res. 2013;67:60–67. 23098819 10.1016/j.phrs.2012.10.010

[16] Xu S, Gao Y, Zhang Q, et al. SIRT1/3 Activation by Resveratrol Attenuates Acute Kidney Injury in a Septic Rat Model. Oxid Med Cell Longev. 2016;2016:7296092 28003866 10.1155/2016/7296092 PMC5149703

[17] Zeng Z, Chen Z, Xu S, et al. Polydatin Protecting Kidneys against Hemorrhagic Shock-Induced Mitochondrial Dysfunction via SIRT1 Activation and p53 Deacetylation. Oxid Med Cell Longev. 2016;2016:1. 10.1155/2016/1737185 PMC4783550 27057271

[18] Zhang W, Huang Q, Zeng Z, et al. Sirt1 Inhibits Oxidative Stress in Vascular Endothelial Cells. Oxid Med Cell Longev. 2017;2017:7543973 28546854 10.1155/2017/7543973 PMC5435972

[19] Chang JW, Kim H, Baek CH, et al. Up-Regulation of SIRT1 Reduces Endoplasmic Reticulum Stress and Renal Fibrosis. Nephron. 2016;133:116–128. 27255945 10.1159/000447067

[20] Xiao Z, Chen C, Meng T, et al. Resveratrol attenuates renal injury and fibrosis by inhibiting transforming growth factor-beta pathway on matrix metalloproteinase 7. Exp Biol Med (Maywood). 2016;241:140–146. 26316584 10.1177/1535370215598401 PMC4935387

[21] Shimoyama Y, Suzuki K, Hamajima N, et al. Sirtuin 1 gene polymorphisms are associated with body fat and blood pressure in Japanese. Transl Res. 2011;157:339–347. 21575918 10.1016/j.trsl.2011.02.004

[22] Kalemci S, Edgunlu TG, Kara M, et al. Sirtuin gene polymorphisms are associated with chronic obstructive pulmonary disease in patients in Muğla province. Kitp. 2014;3:306–310. 10.5114/kitp.2014.45682 PMC4283875 26336440

[23] Hou J, Chen W, Lu H, et al. Exploring the Therapeutic Mechanism of Desmodium styracifolium on Oxalate Crystal-Induced Kidney Injuries Using Comprehensive Approaches Based on Proteomics and Network Pharmacology. Frontiers in Pharmacology. 2018;9:620. 29950996 10.3389/fphar.2018.00620 PMC6008405

[24] Izmirli M, Goktekin O, Bacaksiz A, et al. The effect of the SIRT1 2827 A > G polymorphism, resveratrol, exercise, age and occupation in Turkish population with cardiovascular disease. Anadolu Kardiyol Derg. 2015;15:103–106. 10.5152/akd.2014.5214 PMC5336992 25252293

[25] Nogueiras R, Habegger KM, Chaudhary N, et al. Sirtuin 1 and sirtuin 3: physiological modulators of metabolism. Physiol Rev. 2012;92:1479–1514. 22811431 10.1152/physrev.00022.2011 PMC3746174

[26] Fagerberg L, Hallstrom BM, Oksvold P, et al. Analysis of the human tissue-specific expression by genome-wide integration of transcriptomics and antibody-based proteomics. Mol Cell Proteomics. 2014;13:397–406. 24309898 10.1074/mcp.M113.035600 PMC3916642

[27] Ren Y, Du C, Shi Y, et al. The Sirt1 activator, SRT1720, attenuates renal fibrosis by inhibiting CTGF and oxidative stress. Int J Mol Med. 2017;39:1317–1324. 28339034 10.3892/ijmm.2017.2931

[28] He W, Wang Y, Zhang MZ, et al. Sirt1 activation protects the mouse renal medulla from oxidative injury. J Clin Invest. 2010;120:1056–1068. 20335659 10.1172/JCI41563 PMC2846063

[29] Du YG, Zhang KN, Gao ZL, et al Tangshen formula improves inflammation in renal tissue of diabetic nephropathy through SIRT1/NF-κB pathway. Exp Ther Med. 2018;15:2156–2164. 29434819 10.3892/etm.2017.5621 PMC5776509

[30] Theendakara V, Patent A, Peters Libeu CA, et al. Neuroprotective Sirtuin ratio reversed by ApoE4. Proc Natl Acad Sci USA. 2013;110:18303–18308. 24145446 10.1073/pnas.1314145110 PMC3831497

[31] Hong W, Xu XY, Qiu ZH, et al. Sirt1 is involved in decreased bone formation in aged apolipoprotein E-deficient mice. Acta Pharmacol Sin. 2015;36:1487–1496. 26592520 10.1038/aps.2015.95 PMC4816239

[32] Higashibata T, Wakai K, Naito M, et al. Effects of self-reported calorie restriction on correlations between SIRT1 polymorphisms and body mass index and long-term weight change. Gene. 2016;594:16–22. 27591970 10.1016/j.gene.2016.08.051

[33] Garcia-Chapa EG, Leal-Ugarte E, Peralta-Leal V, et al. Genetic Epidemiology of Type 2 Diabetes in Mexican Mestizos. Biomed Res Int. 2017;2017:3937893 28607931 10.1155/2017/3937893 PMC5451767

[34] Winer DA, Winer S, Dranse HJ, et al. Immunologic impact of the intestine in metabolic disease. J Clin Invest. 2017;127:33–42. 28045403 10.1172/JCI88879 PMC5199708

[35] Kohjimoto Y, Sasaki Y, Iguchi M, et al. Association of metabolic syndrome traits and severity of kidney stones: results from a nationwide survey on urolithiasis in Japan. Am J Kidney Dis. 2013;61:923–929. 23433467 10.1053/j.ajkd.2012.12.028

[36] Khaleel A, Wu MS, Wong HS, et al. A Single Nucleotide Polymorphism (rs4236480) in TRPV5 Calcium Channel Gene Is Associated with Stone Multiplicity in Calcium Nephrolithiasis Patients. Mediators Inflamm. 2015;2015:1. 10.1155/2015/375427 PMC4452106 26089600

[37] Mitra P, Guha M, Ghosh S, et al. Association of calcitonin receptor gene (CALCR) polymorphism with kidney stone disease in the population of West Bengal, India. Gene. 2017;622:23–28. 28435134 10.1016/j.gene.2017.04.033

[38] Goknar N, ktem F, Torun E, et al. The role of vitamin D receptor gene polymorphisms in Turkish infants with urolithiasis. Ren Fail. 2016;38:545–551. 26908058 10.3109/0886022X.2016.1148557

[39] Zainabadi K, Liu CJ, Guarente L SIRT1 is a positive regulator of the master osteoblast transcription factor, RUNX2. PLoS One. 2017;12:e0178520. 28542607 10.1371/journal.pone.0178520 PMC5444833

[40] Gong K, Qu B, Wang C, et al. Peroxisome Proliferator-Activated Receptor alpha Facilitates Osteogenic Differentiation in MC3T3-E1 Cells via the Sirtuin 1-Dependent Signaling Pathway. Mol Cells 2017;40:393–400. 28614912 10.14348/molcells.2017.0018 PMC5523015

[41] Coe FL, Worcester EM, Evan AP Idiopathic hypercalciuria and formation of calcium renal stones. Nat Rev Nephrol. 2016;12:519–533. 27452364 10.1038/nrneph.2016.101 PMC5837277

[42] Clark SJ, Falchi M, Olsson B, et al. Association of sirtuin 1 (SIRT1) gene SNPs and transcript expression levels with severe obesity. Obesity (Silver Spring). 2012;20:178–185. 21760635 10.1038/oby.2011.200 PMC3760128

[43] Zheng J, Chen LL, Xiao F, et al. Three single nucleotide variants of the SIRT1 gene are associated with overweight in a Chinese population: a case control study. Endocr J. 2012;59:229–237. 22230810 10.1507/endocrj.ej11-0234

[44] Botden IP, Zillikens MC, de Rooij SR, et al. Variants in the SIRT1 gene may affect diabetes risk in interaction with prenatal exposure to famine. Diabetes Care. 2012;35:424–426. 22228742 10.2337/dc11-1203 PMC3263901

[45] Han J, Wei M, Wang Q, et al. Association of Genetic Variants of SIRT1 With Type 2 Diabetes Mellitus. Gene Expr. 2015;16:177–185. 26637398 10.3727/105221615X14399878166195 PMC8750030

[46] Rizk SM, Shahin NN, Shaker OG Association between SIRT1 Gene Polymorphisms and Breast Cancer in Egyptians. PLoS One. 2016;11:e0151901 26999517 10.1371/journal.pone.0151901 PMC4801365

[47] Wang XL, Wu LY, Zhao L, et al. SIRT1 activator ameliorates the renal tubular injury induced by hyperglycemia in vivo and in vitro via inhibiting apoptosis. Biomed Pharmacother. 2016;83:41–50. 27470548 10.1016/j.biopha.2016.06.009

[48] Huang J, Sun L, Liu M, et al. Association between SIRT1 gene polymorphisms and longevity of populations from Yongfu region of Guangxi. Zhonghua Yi Xue Yi Chuan Xue Za Zhi. 2013;30:55–59. 23450480 10.3760/cma.j.issn.1003-9406.2013.01.013

[49] Han Q, Zhang Y, Li W, et al. Functional sequence variants within the SIRT1 gene promoter in indirect inguinal hernia. Gene. 2014;546:1–5. 24875419 10.1016/j.gene.2014.05.058

